# Editorial: Integrative multi-modal, multi-omics analytics for the better understanding of metabolic diseases

**DOI:** 10.3389/fendo.2023.1266557

**Published:** 2023-09-08

**Authors:** Animesh Acharjee, Prasoon Agarwal, Georgios V. Gkoutos

**Affiliations:** ^1^ College of Medical and Dental Sciences, Institute of Cancer and Genomic Sciences, University of Birmingham, Birmingham, United Kingdom; ^2^ Institute of Translational Medicine, University Hospitals Birmingham National Health Service (NHS) Foundation Trust, Birmingham, United Kingdom; ^3^ Medical Research Council (MRC) Health Data Research United Kingdom (UK) (HDR), Midlands Site, Birmingham, United Kingdom; ^4^ Centre for Health Data Research , University of Birmingham, Birmingham, United Kingdom; ^5^ National Bioinformatics Infrastructure Sweden (NBIS), Science for Life Laboratory, Division of Occupational and Environmental Medicine, Department of Laboratory Medicine, Lund University, Lund, Sweden; ^6^ Cancer Research, National Institute for Health and Care Research (NIHR) Experimental Cancer Medicine Centre, Birmingham, United Kingdom

**Keywords:** omics, diagnostic, biomarker, therapeutic, metabolic disease

In the past few years, large-scale, high-throughput multi-omics experiments and improved clinical measurements have led to the generation of a plethora of multi-modal datasets related to various metabolic diseases (MetS), for example, type 1 diabetes (T1D), obesity, non-alcoholic fatty liver disease (NAFLD), etc. Many integration strategies have been discussed in the literature, such as early, intermediate, and late integration (1). The process of early integration involves merging multiple omics information sources into a unified matrix, whereas intermediate integration involves transforming the source datasets into representations that are both common and specific to omics. Late integration consists of the individual analysis of each omics dataset, followed by the combination of their respective predictions to obtain a result (1). **Figure 1** shows an example of late integration described in the context of MetS.

**Figure 1:**
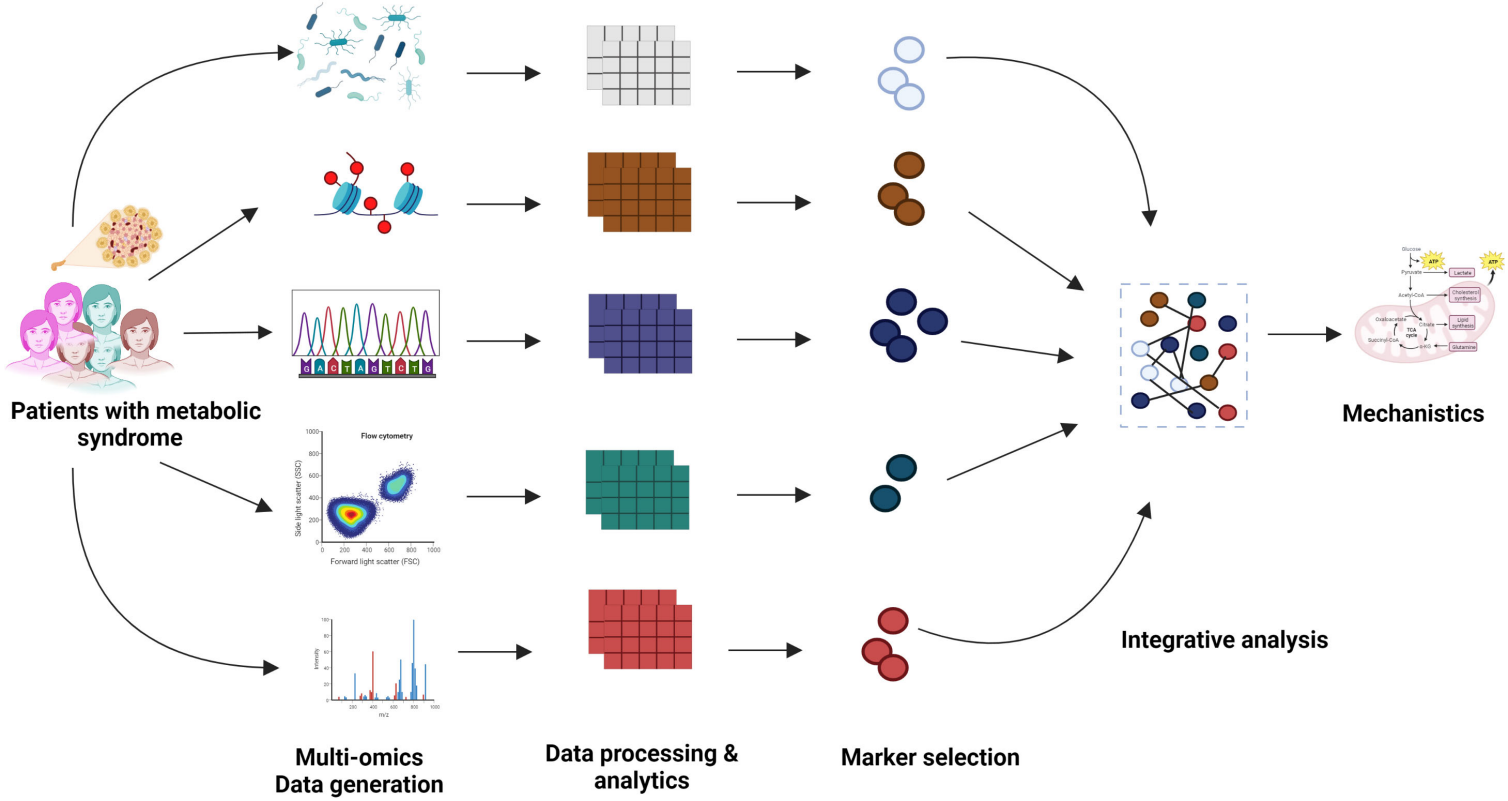
Graphical representation of the multi-modal integration to understand the mechanistic aspects of the metabolic disease.

This editorial summarizes the contributions to the Research Topic of Frontiers in Endocrinology, “*Integrative Multi-Modal, Multi-Omics Analytics for the Better Understanding of Metabolic Diseases*”, between November 2021 and July 2023. This Research Topic aims to provide a platform for researchers in multi-omics and multi-modal analysis of metabolic diseases to identify targets for therapy and diagnosis. A total of 10 original articles were selected for publication from the submissions received. A summary of each manuscript is detailed below.

We have grouped the papers based on the different types of diseases they address, starting with hyperlipidemia, which is characterized by elevated levels of lipids, for example, cholesterol and triglycerides. The study performed by Zhai et al. investigated the effects of policosanol on the control of hyperlipidemia, gut microbiome composition, and metabolic status using a C57BL/6 mouse model. Serum triglycerides, total cholesterol, and brown adipose tissue weight were all shown to be dramatically reduced after policosanol administration. Overall, policosanol demonstrated to the potential to alter the makeup of the gut microbiota, speed up fat breakdown, and differentiate thermogenesis-related gene activity.

It has been noted that vitamin D also affects patients with metabolic syndrome, and Amirkhizi et al. conducted a case-control study to investigate the relationship between vitamin D deficiency and adipokines, atherogenesis indicators, and metabolic syndrome factors in 195 patients with metabolic syndrome. The study demonstrated that patients with MetS and vitamin D insufficiency (cases) had higher AIP and LAP than controls. Moreover, vitamin D deficiency was correlated with some of the cardiometabolic risk factors in patients with MetS.

T1D is characterized by multiple factors, i.e., genetics, lifestyle, and metabolism. Clos-Garcia et al. investigated the gut microbiome and blood metabolome in individuals with T1D and healthy controls. The study stratified T1D cases based on albuminuria levels, identifying 51 species with and without albuminuria. Plasma metabolomics analysis identified differences in steroidogenesis, glucose metabolism, and circulating sphingolipids in subjects with T1D. Furthermore, the analysis revealed reduced interactions between the gut microbiome and plasma metabolome profiles, while polar metabolite, lipid, and bacteriome compositions contributed to the variance in albuminuria levels among T1D individuals. Zhang et al. analyzed the serum metabolic profiles of children with T1D diabetes and healthy controls and identified many differential metabolites, such as carbohydrates, indoles, unsaturated fatty acids, amino acids, and organic acids, consistently across pediatric patients.

The transcriptome and proteome also play a role in MetS, for example, in patients with non-alcoholic steatohepatitis (NASH). Pyo and Choi found that upregulated genes were associated with inflammation, steatosis, apoptosis, and extracellular matrix organization, while downregulated genes were associated with the response to metal ions and lipid and amino acid metabolism. Functional enrichment analysis revealed amino acid metabolism as the most significant hepatic perturbation in both human and mouse NASH.

Wang et al. developed a new obesity measurement index for metabolically associated fatty liver disease (MAFLD), which includes traditional BMI. The results showed a strong correlation between MAFLD, traditional BMI, and the new index.

Epithelial-mesenchymal transition (EMT) is a critical event in the migration and invasion of endometriosis, involving immune and stromal cells. Quan et al. investigated the potential use of EMT-based classification for the precise diagnosis and treatment of peritoneal endometriosis. A total of 76 peritoneal endometriosis samples were classified into two clusters based on EMT hallmark genes. EMT scores and abundances were compared, and a diagnostic model was constructed based on 9 markers related to immune and stromal scores.

Pan et al. developed a targeted method for the accurate quantification of 80 bile acids in gastric cancer patients with the aim of developing diagnostics for the early screening of GC. The panel of six bile acids (ratio), which included HCA, TLCA, NorCA, DCA-3G, TLCA-3S, and HDCA/LCA, showed high accuracy for the diagnosis of gastric cancer.

Yuan et al. investigated the causal relationship between rheumatoid arthritis (RA) and atlantoaxial subluxation (AAS), identifying and quantifying the potential involvement of C-reactive protein as a mediator. The researchers utilized a genome-wide association study, a two-sample Mendelian randomization (MR) analysis of genetically predicted rheumatoid arthritis, and an AAS. The MR analysis revealed a higher genetically predicted risk of rheumatoid arthritis and an increased risk of AAS. There was no convincing evidence that genetically predicted AAS affected the risk of rheumatoid arthritis.

Pirzada et al. used machine learning to discover potential inhibitors of GSK3, a protein involved in the replication and assembly of the nucleocapsid protein of SARS-CoV-2 and other coronaviruses. They used a dataset of FDA-approved and investigational pharmaceuticals from the ChEMBL database and a variety of molecular descriptors to define the inhibitors. Based on their predicted activity, selinexor and ruboxistaurin were identified as the two most promising candidates. The study demonstrated that this virtual high-throughput screening approach based on artificial intelligence can accelerate drug discovery and identify novel targets.

In summary, this Research Topic presented multiple MetS-related studies. Multi-omics and multi-modal datasets, such as clinical data (Clos-Garcia et al.), transcriptomics (Pyo and Choi), metabolomics (Clos-Garcia et al.), and gut microbiome datasets (Clos-Garcia et al.), have been utilized to understand metabolic diseases. Thus, the results of these studies improve the prospects for future therapeutic and translational studies (2).

## Author contributions

AA: Conceptualization, Funding acquisition, Investigation, Supervision, Visualization, Writing – original draft, Writing – review & editing, Formal Analysis, Methodology, Validation. PA: Conceptualization, Data curation, Investigation, Writing – original draft, Writing – review & editing. GG: Conceptualization, Funding acquisition, Investigation, Writing – original draft, Writing – review & editing.
